# Suboptimal Coding Metasurfaces for Terahertz Diffuse Scattering

**DOI:** 10.1038/s41598-018-30375-z

**Published:** 2018-08-09

**Authors:** Massimo Moccia, Can Koral, Gian Paolo Papari, Shuo Liu, Lei Zhang, Rui Yuan Wu, Giuseppe Castaldi, Tie Jun Cui, Vincenzo Galdi, Antonello Andreone

**Affiliations:** 1 Fields & Waves Lab, Department of Engineering, University of Sannio, I-82100 Benevento, Italy; 2 Department of Physics, University of Naples “Federico II”, and CNR-SPIN, I-80125 Naples, Italy; 3 INFN Naples Unit, via Cinthia, I-80126 Naples, Italy; 4 State Key Laboratory of Millimeter Waves, Southeast University, Nanjing, 210096 China

## Abstract

Coding metasurfaces, composed of only two types of elements arranged according to a binary code, are attracting a steadily increasing interest in many application scenarios. In this study, we apply this concept to attain diffuse scattering at THz frequencies. Building up on previously derived theoretical results, we carry out a suboptimal metasurface design based on a simple, deterministic and computationally inexpensive algorithm that can be applied to arbitrarily large structures. For experimental validation, we fabricate and characterize three prototypes working at 1 THz, which, in accordance with numerical predictions, exhibit significant reductions of the radar cross-section, with reasonably good frequency and angular stability. Besides the radar-signature control, our results may also find potentially interesting applications to diffusive imaging, computational imaging, and (scaled to optical wavelengths) photovoltaics.

## Introduction

Metamaterials and metasurfaces^1–3^ are artificial materials composed of (3-D and 2-D, respectively) arrangements of sub-wavelength inclusions, which are engineered so as to tailor the effective properties in a precise, desired fashion, not necessarily attainable in conventional materials. For instance, by relying on powerful design approaches such as *transformation optics*^4^, a desired field manipulation can be engineered via a prescribed local tailoring of the constitutive parameters in the region of interest. On the other hand, abrupt changes over the wavelength scale in the phase, amplitude and/or polarization of a wavefront can be impressed via ultrathin *gradient* metasurfaces^5^, thereby extending the conventional Snell’s reflection and refraction laws.

In the above examples, the material properties of the inclusions are primarily constrained by the practical availability and, depending on the application, by power dissipation, whereas the shape of the inclusions is typically retrieved via suitably constrained inverse-design procedures, and can vary across a large parameter space. Recently, the idea of “digitizing” the parameter space of the inclusions, i.e., relying on a limited number of inclusion types, was put forward by Della Giovanpaola and Engheta^6^ and by Cui *et al*^7^. for metamaterials and metasurfaces, respectively. Within this framework, particularly intriguing appear the so-called “coding metasurfaces”, in which a binary code is associated with each possible inclusion (unit cell). Over the past few years, these structures have been the subject of intense investigation (see, e.g., refs^8–25^), and their applications have been recently extended also to the acoustic domain^26,27^. The reader is also referred to refs^28,29^ for recent comprehensive reviews on the subject, which also explore the fascinating concepts of “programmable” and “information-based” metasurfaces.

Of particular interest for the present study are the applications to *diffuse scattering* at THz frequencies^9–12,14,15,22,23^. Compared with microwave frequencies, the study of metasurfaces in the THz region might be of benefit for a number of different applications, including imaging, radar, and sensing, in view of the inherent higher spatial resolution, stronger secrecy, penetration capability, interference immunity, and field enhancement. In particular, as THz imaging radars^30,31^ are becoming increasingly common in security and safety applications, the design of low-scattering fixtures is gaining a growing attention. Diffuse-scattering metasurfaces appear to be an attractive solution in view of their potential conformability (by relying on flexible substrates) as well as the negligible impact on the thermal signature (as opposed, e.g., to absorbers). Besides the reduction/control of the radar signature, they may also find important potential applications to diffusive imaging^32^, computational imaging^13,33^, and (at optical wavelengths) to light trapping in photovoltaics^34^.

The design of a coding metasurface for diffuse scattering represents a fairly complex combinatorial problem, typically addressed via computationally intensive optimization algorithms^11,20,35^, which do not guarantee finding the global optimum, and may become unaffordable for electrically large structures. In a recent study^25^, we proposed a sub-optimal, computationally inexpensive design procedure based on a class of *flat* polynomials^36–38^. Here, we apply this approach to the design of coding metasurfaces for diffuse scattering at THz frequencies, and we experimentally validate it via time-domain-spectroscopy measurements on fabricated prototypes operating at 1 THz.

## Results

**Figure 1:**
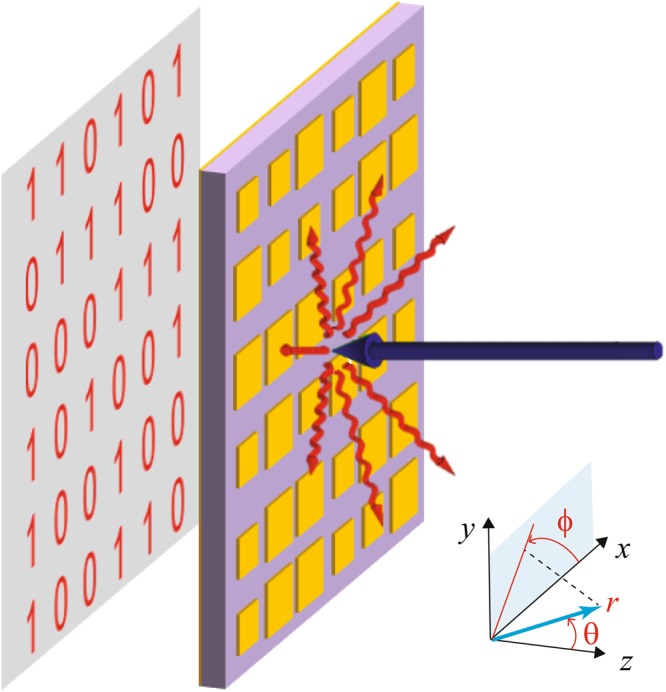
Problem schematic. A binary-coded metasurface (yellow-purple pattern) made of only two types of unit-cells yields diffuse scattering under normally incident plane-wave illumination. The binary coding is displayed behind. Also shown are the relevant Cartesian (*x*, *y*, *z*) and associated spherical (*r*, *θ*, *ϕ*) reference systems.

The basic idea is schematized in Fig. 1. We consider a metasurface composed of a metal-backed dielectric film patterned with two types of unit cells made of metallic-patch elements (labeled as “1” and “0”) arranged according to a 2-D binary coding. We assume that the metasurface is placed in the *x*-*y* plane of a Cartesian reference system (see Fig. 1) and, unless otherwise specified, that a plane wave is normally impinging along the (negative) *z*-axis, with *y*-polarized electric field. Our aim is to engineer the unit cells and the coding in order to attain *diffuse scattering*, i.e., to scatter the impinging plane wave in all possible directions, as uniformly as possible, so as to avoid the emergence of strong Bragg-type peaks.

As previously mentioned, this problem is typically addressed via brute-force numerical optimization^11,20,35^. Instead, here we follow a different, recently proposed approach^25^ that directly exploits the spectral properties of certain aperiodic binary sequences known as Golay-Rudin-Shapiro (GRS) sequences^36–38^. These sequences are characterized by an *absolutely continuous* spectral response (which is representative of the desired diffuse-scattering response), and a so-called “trivial” Bragg spectrum that can be suppressed by suitably tailoring the scattering response of the two basic unit-cells^39^. In particular, if multiple scattering is neglected, such suppression is attained by choosing the two unit-cell scattering responses equal in magnitude and 180° out of phase^39^.

**Figure 2:**
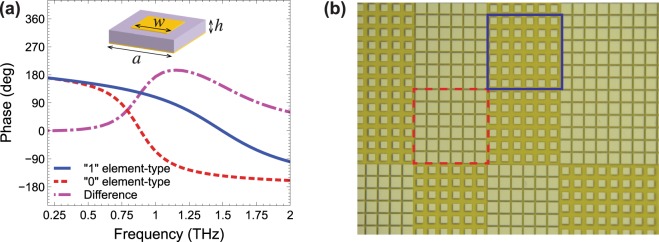
(**a**) Numerically computed reflection-coefficient phase as a function of frequency, pertaining to the “1” (blue-solid curve) and “0” (red-dashed curve) element types, assuming normally incident plane-wave illumination with electric field parallel to the patch side. Also shown (magenta-dashed-dotted curve) is the phase difference between the two configurations (which becomes 180° at the design frequency of 1 THz). An infinite periodic structure is assumed, composed of unit cells (see inset) of period *a* = 50 μm, with patch sidelength *w*_1_ = 30 μm and *w*_0_ = 45 μm, for the “1” and “0” element-types, respectively; a substrate of thickness *h* = 20 μm is assumed, with *ε*_*r*_ = 3, $\tan \,\delta =0.01$, and perfectly electric conducting metallization. (**b**) Detailed view of an optical microscope image of a fabricated sample, with the 6 × 6 supercells pertaining to the “1” and “0” element-types identified with blue-solid and red-dashed squares, respectively.

As shown in Fig. 2a, in the chosen configuration the above out-of-phase condition can be engineered at a desired frequency (1 THz, in our case) by suitably choosing the patch size of the two elements.

The binary coding is generated via a simple deterministic algorithm in two steps. First, an auxiliary binary sequence is generated with the alphabet {−1, 1},

1 $${\chi }_{0}=1,{\chi }_{2n}={\chi }_{n},{\chi }_{2n+1}={(-1)}^{n},$$

and is mapped onto the {0, 1} alphabet as follows:

2 $${\alpha }_{n}=\frac{1+{\chi }_{n}}{2}.$$

The 2-D coding is finally obtained via dyadic product of the 1-D sequence {*α*_*n*_} by itself. In this study, we consider a sequence length *N* = 2^*ν*^(with *ν* = 1, 2, ...), for which the above coding is strictly related to the so-called GRS polynomials^36–38^, which are known to exhibit some spectral *flatness* properties that are especially desirable for our diffuse-scattering application (see also the discussion in ref.^25^). More specifically, there are two types of GRS polynomials (henceforth referred to as *P*-type and *Q*-type), which can be explicitly defined via two intertwined recursive relationships^36–38^

3 $$\begin{array}{c}{P}_{\nu +1}(\xi )={P}_{\nu }(\xi )+{\xi }^{{2}^{\nu }}{Q}_{\nu }(\xi ),\\ {Q}_{\nu +1}(\xi )={P}_{\nu }(\xi )-{\xi }^{{2}^{\nu }}{Q}_{\nu }(\xi ),\end{array}$$

initialized by *P*_0_ = *Q*_0_ = 1, and with *ξ* denoting a complex-valued (generally unimodular) variable. It can be shown^36–38^, that the coefficients of the GRS polynomials in Eq. (3) are ±1. In particular, the coefficients of the *P*_*ν*_-type GRS polynomials are given by the coding sequence {*χ*_*n*_} in Eq. (1), whereas those pertaining to the *Q*_*ν*_-type GRS polynomials are obtained by flipping the bit elements in the second half of the sequence {*χ*_*n*_}^36–38^. In ref.^25^, we demonstrated that these designs are *suboptimal*, in the sense that the attainable radar-cross-section (RCS) reduction (by comparison with an unpatterned metallic target of same size) is only few dB distant from that obtained via computationally-intensive numerical optimization^11,20,35^ as well as from theoretically derived tight bounds. In what follows, we provide further numerical evidence of this suboptimal character.

Based on the above algorithm, we design and fabricate three metasurface prototypes, with the coding chosen according to the *P*_2_, *P*_5_, and *Q*_5_ GRS polynomials. The coding sequences and corresponding patterns are explicitly given in the Supplementary Information (Table S1, Fig. S1). Specifically, the GRS *P*_5_- and *Q*_5_-type codings consist of 32 × 32 bit elements, whereas the *P*_2_-type coding consists only of 4 × 4 elements, and is replicated 8 × 8 times in order to cover the same area as the other two. In view of this long-range periodicity, the *P*_2_-type coding metasurface is not particularly suited for diffuse scattering but, as it will be clear hereafter, it is mainly considered for calibration purposes.

Figure 2b displays a detailed view of an optical microscope image of a fabricated sample. Note that each bit element is actually a “supercell” made of several (6 × 6, in our case) elementary unit cells, so as to establish a local periodicity on the scale of a wavelength. This is necessary in order to ensure the self-consistency of the model, since the physical unit cells are designed via full-wave simulations assuming infinite periodic structures (see ref.^25^ for more details).

For the experimental characterization, we utilize a time-domain spectroscopy system. More specifically, with the experimental setups detailed in the Supplementary Information (see the schematics in Fig. S2 and renderings in Fig. S3), we measure the scattered field intensity *I*_*MS*_ from the metasurface as a function of the frequency *f* and direction *θ* (in the *ϕ* = 0 plane parallel to the patch side; cf. Fig. 1). As a reference, we also measure the intensity *I*_*metal*_ reflected from an unpatterned metal region of same size, and calculate the RCS ratio

4 $$\gamma (f,\theta )=\frac{{I}_{MS}(f,\theta )}{{I}_{metal}(f,{\theta }_{s})},$$

with *θ*_*s*_ denoting the specular-reflection direction (at which the reflected-intensity from the unpatterned metal reference is maximum).

**Figure 3:**
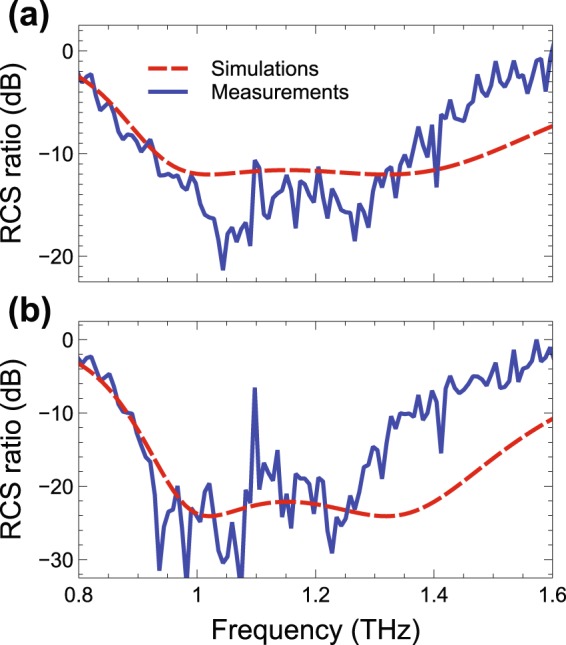
(**a**,**b**) Measured (blue-solid) and simulated (red-dashed) RCS ratio [Eq. (4)] for normal-incidence and backscattering-direction (*θ* = *θ*_*i*_ = 0, obtained with the setup in Fig. S2a in the Supplementary Information), as a function of frequency, for the GRS *P*_2_- and *P*_5_-type designs, respectively. The coding sequences and corresponding layouts are explicitly given in Table S1 and Fig. S1 in the Supplementary Information.

Figure 3 shows the results for normal-incidence and backscattering-direction (*θ* = *θ*_*i*_ = 0, with electric field parallel to the patch side, obtained with the setup in Fig. S2a in the Supplementary Information), as a function of frequency, for the *P*_2_- and *P*_5_-type prototypes. Note that the metasurface size is 9.6 × 9.6 mm^2^ (i.e., ~32 wavelengths per linear dimension at the design frequency of 1 THz), and a full-wave simulation of the entire structures is unaffordable with our current computational resources. Accordingly, we utilize instead the semi-analytical model detailed in ref.^25^ (see also the Methods section below for more details). Such model, based on a physical-optics approximation, has been shown to provide a good agreement with full-wave simulations and measurements^25^. Also in the present case, as it can be observed from Fig. 3, the agreement between measurements and simulations is fairly good, and indicates a significant reduction of the RCS (~10 dB and ~20 dB, for the *P*_2_- and *P*_5_-type, respectively), over a sizable frequency region around 1 THz. The deterioration of the agreement at higher frequencies is attributable to the various approximations and unmodeled effects in the simulations, as well as uncertainties in the parameters and fabrication tolerances. The rather strong reflection peak around 1.1 THz (especially visible in the response of the *P*_5_-type sample in Fig. 3b) is attributable to water-vapor absorption (see also the discussion below).

**Figure 4:**
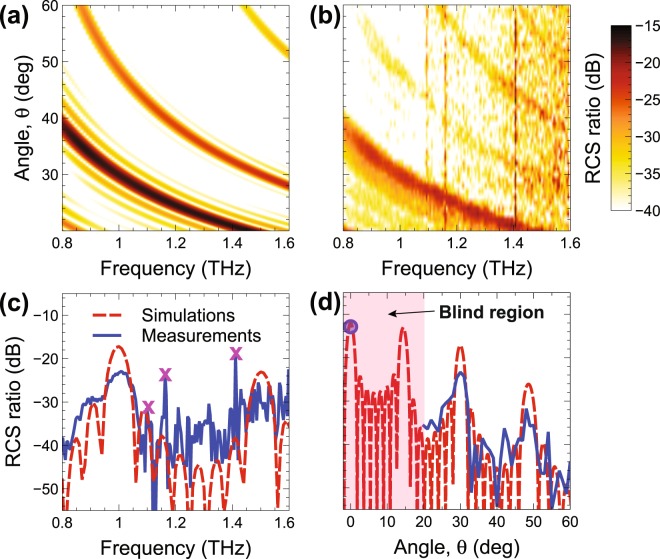
(**a**,**b**) Simulated and measured, respectively, RCS ratio [Eq. (4)] in false-color scale, for normal-incidence, as a function of frequency and observation angle, for the GRS *P*_2_-type design. Measurements are obtained via the setup in Fig. S2b in the Supplementary Information. (**c**,**d**) Representative cuts at *θ* = 30° and *f* = 1 THz, respectively, comparing measurements (blue-solid) and simulations (red-dashed). The magenta-cross markers in panel (c) indicate the water-vapor absorption peaks. The pink-shaded area in panel (d) indicates a 20° angular region that is not accessible by the detector (blind region, cf. Fig. S2b). Also shown (blue-circle marker) is the backscattering measurement sample (extracted from Fig. 3a).

The anticipated poorer performance of the *P*_2_-type design can be understood from the results in Fig. 4. More specifically, the false-color-scale maps in Fig. 4a and b show the simulated and measured (via the setup in Fig. S2b in the Supplementary Information), respectively, RCS ratio for normal incidence, as a function of the observation angle and frequency, whereas Fig. 4c and show two representative frequency and angular cuts, respectively. Once again, a generally good agreement between simulations and measurements is observed. In view of the replication-induced long-range periodicity, the *P*_2_-type coding metasurface exhibits a Bragg-type spectrum with rather sharp peaks, which obviously implies quite poor performance as a diffusive scatterer. In fact, this design mainly serves for calibration purposes, in order to demonstrate that our measurement setup is actually capable of capturing sharp Bragg-type peaks whenever present. As it can be observed from Fig. 4d, both Bragg peaks appearing in the accessible region are captured by our measurements. Also shown is the backscattering measurement sample (extracted from Fig. 3a), which falls within the blind region inaccessible by the detector (see Fig. S2b in the Supplementary Information), and is once again in good agreement with the numerical prediction. Note that, in the measured map (Fig. 4b), the distinctive angle-independent scattering features around the frequencies of 1.1, 1.15, and 1.4 THz correspond to well-known water-vapor absorption peaks^40^. Moreover, the presence of a modal branch that does not appear in the simulated map (Fig. 4a) is likely attributable to the imperfect suppression of a trivial Bragg-mode^39^.

**Figure 5:**
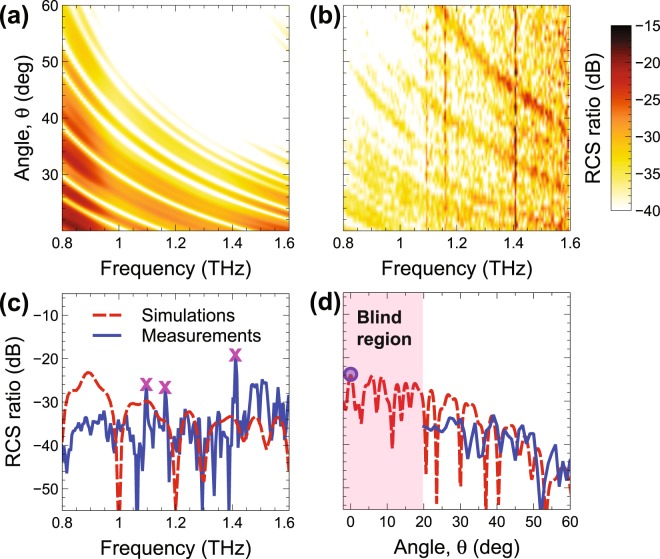
As in Fig. 4, but for the GRS *P*_5_-type design.

Figure 5 shows the corresponding results for the *P*_5_-type design. In this case, the structure does not exhibit long-range order, and this yields a rather *flat* response, devoid of Bragg-peaks, in line with the desired diffuse-scattering behavior.

**Figure 6:**
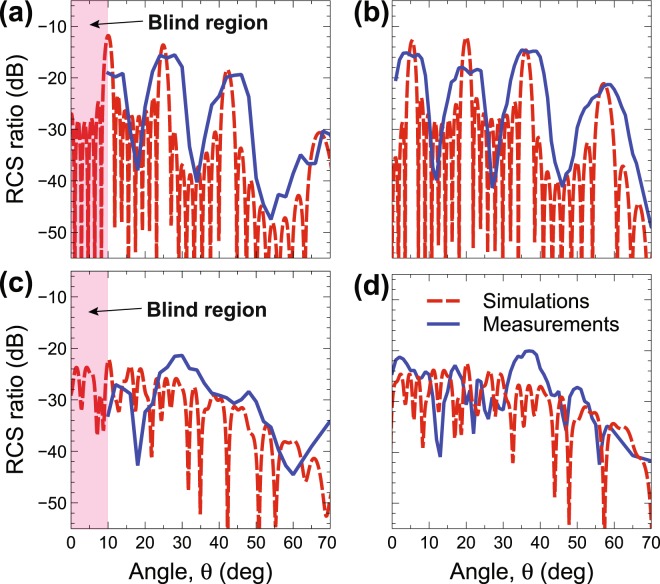
(**a**,**b**) As in Fig. 4d (GRS *P*_2_-type design), but for oblique incidence with *θ*_*i*_ = 10° and 20°, respectively. (**c**,**d**) As in Fig. 5d (GRS *P*_5_-type design) but for oblique incidence with *θ*_*i*_ = 10° and 20°, respectively. Note that, for the chosen observation ranges, the blind region is 10° for *θ*_*i*_ = 10° and does not occur for *θ*_*i*_ = 20°.

Although the metasurfaces are designed to work at normal incidence, it is interesting to study their sensitivity to oblique-incidence conditions. Figure 6 illustrates, for the *P*_2_- and *P*_5_-type designs, the results for oblique incidence of 10° and 20° (while maintaining the electric field parallel to the patch side), at the design frequency of 1 THz. Once again, measurements and simulations are in reasonably good agreement, and the Bragg-peaks appearing in the *P*_2_-type case (Fig. 6a and b) are accurately captured in position and magnitude. Once again, the broader character of the measured peaks (by comparison with the simulated ones) can be attributed to the various approximations and unmodeled effects in the numerical simulations. Prominent among them are the finite-size of the impinging beam (assumed as a plane wave in the simulations) and the element-coupling (multiple scattering) effects. In connection with the *P*_5_-type design (Fig. 6c and d), we remark the absence of strong spectral features, which is indicative of a rather good angular stability.

Results qualitatively similar to the *P*_5_-type design are also observed for the *Q*_5_-type case, as shown in Figs S4–S6 in the Supplementary Information.

In order to quantitatively assess the optimality of the proposed design, a meaningful observable is the (worst-case) RCS ratio for normal incidence

5 $$\hat{\gamma }=\frac{\mathop{{\rm{\max }}}\limits_{\theta ,\varphi }{I}_{MS}(f,\theta ,\varphi )}{{I}_{metal}(f,\theta =0)},$$

which represents the maximum scattering intensity (along all possible directions) normalized with respect to that backscattered by an unpatterned metallic surface of same size. Clearly, the smaller this value, the more effective the coding metasurface in attaining diffuse scattering.

By assuming a metasurface composed of *N* × *N* square supercells of sidelength *d*, and applying the semi-analytical modeling detailed in ref.^25^, in view of the well-known *spectral-flatness* properties^36–38^ of the GRS polynomials, it can be shown^25^ that the maximum RCS ratio in Eq. (5) approximately scales as

6 $${\hat{\gamma }}_{GRS}\sim C{(\frac{\lambda }{Nd})}^{2},$$

where *C* is a constant essentially dependent on the supercell size, and *λ* = *c*/*f* is the vacuum wavelength (with *c* denoting the corresponding wavespeed).

As previously mentioned, a brute-force numerical optimization of the coding pattern, so as to minimize the RCS ratio in Eq. (5), is only possible for moderately sized structures, but may become computationally unaffordable for electrically large structures. Moreover, given the inherently nonquadratic character of the optimization problem, numerical algorithms tend to be prone to false solutions, and thus there is no guarantee to attain a global minimum. In ref.^7^, a hybrid numerical optimization approach was exploited for structures of (linear) electrical size up to ~20*λ*. In ref.^25^, via a numerical fit, we showed that the scaling law of the RCS ratio pertaining to these optimized structures was qualitatively similar to that in Eq. (6). Although the numerical fit was based on electrical sizes smaller than those of interest in the present study, its extrapolation can still be assumed as a meaningful benchmark. Accordingly, in what follows, we consider such empirical scaling law

7 $${\hat{\gamma }}_{NOE}\approx 2.552{(\frac{\lambda }{Nd})}^{2.187},$$

and refer to that as “numerical optimization extrapolation” (NOE).

**Figure 7:**
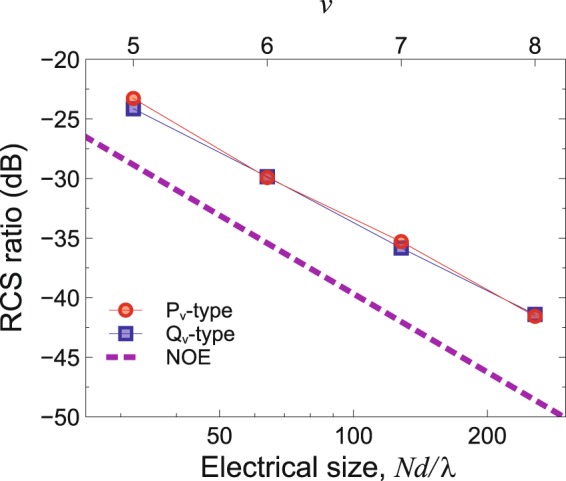
Numerically computed RCS ratio (worst case) in Eq. (5) for the *P*_ν_-type (red-circle markers) and *Q*_ν_-type (blue-square markers) designs as a function of the metasurface electrical size on a log-log scale. Continuous curves are guides to the eye only. The corresponding order ν is shown on the top axis. Geometry and parameters are as in Fig. 2, with supercell size *d* = 6*a* = 300 *μ*m = *λ* (at the operational frequency of 1 THz), and *N* = 2^*ν*^. Also shown (purple-dashed line), as a reference, is the NOE scaling law in Eq. (7).

For the geometry and parameters as in Fig. 2, Fig. 7 shows the numerically computed RCS-ratio scaling laws pertaining to the GRS P-type and Q-type designs, with orders *ν* = 5, 6, 7, 8 (i.e., linear electrical size ranging from 32*λ* to 256*λ*), compared with the NOE prediction in Eq. (7). For both *P*- and *Q*-type GRS coding designs, the log-log scale of the plot highlights an algebraic decay in line with the theoretical predictions in Eqs (6) and (7), with only slight differences on the order of ~5 dB.

We can therefore conclude that our proposed GRS-based coding design is *suboptimal*, in the sense that it provides a RCS reduction that is only slightly worse than the NOE prediction, *irrespective* of the electrical size. However, it is important to stress that, by comparison with brute-force numerical optimization^7^, our *fully deterministic* design approach [essentially relying on Eqs (1) and (2)] requires only a negligible computational effort, and can therefore be applied to *arbitrarily large* structures.

## Discussion

To sum up, we have presented the suboptimal design, fabrication and experimental characterization of coding metasurfaces acting as diffusive scatterers at THz frequencies. Our results extend the experimental validation of our general design approach to the THz band. It is worth pointing out that, while our previous experimental validation at microwave frequencies^25^ was limited to relatively small electrical sizes (about 8 wavelengths per linear dimension) due to inherent limitations of our measurement setup, the THz structures characterized here are considerably larger (32 wavelengths), thereby providing a stronger validation of our proposed design. This is crucially important, as our design approach becomes computationally attractive especially in the limit of electrically large structures.

Overall, our THz study confirms the possibility to effectively design electrically large diffusive scatterers via a simple, deterministic and computationally cheap algorithm, with performance comparable with that attainable via computationally expensive brute-force optimization. Moreover, a reasonably good frequency and angular stability is observed, with ample room for improvement via suitable optimization of the basic unit-cells. Current and future research is aimed at exploring more flexible coding strategies (e.g., multibit) for wideband and wide-angle optimization, as well as possible applications to diffuse imaging and computational imaging. Also of great interest is the extension of these results to optical wavelengths, with possible applications to light trapping in photovoltaics.

## Methods

### Numerical Modeling

The reflection-coefficient phase responses of the unit-cells (Fig. 2a) are obtained via finite-element full-wave simulations, by means of the commercial software package Ansys® HFSS (Electromagnetics suite Release 16.2.0, www.ansys.com/Products/Electronics/ANSYS-HFSS). In these simulations, an infinite structure is assumed, with master/slave periodicity boundary conditions (or phase-shift walls, for oblique incidence) at the four sides of the unit cell, and an air box of thickness 260 μm with a port-type termination on top of the metal patch. The electric field is assumed as parallel to the patch side (i.e., *y*-oriented, in the reference system of Fig. 1). For the dielectric film, a nondispersive model is utilized, with relative permittivity *ε*_*r*_ = 3 and loss-tangent $\tan \,\delta =0.01$, whereas the metal is assumed as perfectly electric conducting. The structure is discretized via the default adaptive meshing (with maximum element size of ~630 nm), which results in about 7,000 degrees of freedom.

For the simulation of the entire metasurfaces, the semi-analytical model detailed in ref.^25^ is utilized, with the single-element response given by the full-wave simulations above.

### Prototype Fabrication

First, a metallic film (10 nm titanium and 200 nm gold) is deposited onto a silicon wafer via electron-beam evaporation. Then, a 20 μm-thick polyimide layer is coated on the gold film, and is baked on a hot plate at 80, 120, 180, and 250 °C for 5 minutes each. Since the liquid polyimide utilized (Yi Dun New Materials Co. Ltd, Suzhou, China) can only be used to form a maximum thickness of 10 μm at the minimum spin rate of 1150 rpm, the above spin-coating and curing processes are repeated twice for the final completion of the 20 μm-thick polyimide layer. To enable a good formation of the metallic pattern during the final lift-off process, a dual-photoresist approach is adopted, which includes a successive coating of LOR-10A and AZ-5214 photoresists, each followed by a soft-bake process on a hotplate. An ultraviolet exposure and develop processes help transfer the mask pattern to the photoresist. Next, another 10/200 nm titanium/gold layer is deposited via electron-beam, followed by an ultrasonic bath in acetone to form the final metallic pattern.

### Measurements

The metasurface characterization is carried out by means of a customized fiber-coupled THz time-domain spectrometer.

For backscattering measurements (Fig. 3), the setup schematized in Fig. S2a (Supporting Information) is utilized, in which the THz beam normally impinges onto the target after passing through a beam-splitter, and the backscattered signal is collected along the orthogonal direction. For angular scattering measurements (Figs 4–6, S5 and S6), the setup in Fig. S2b (Supporting Information) is utilized, in which the THz beam directly impinges onto the target (normally or obliquely), and the scattered signal is measured along a circular path, with the detector positioned on a goniometer. In all measurements, the electric field is maintained parallel to the patch side (i.e., *y*-oriented in the reference system of Fig. 1).

Due to the physical size of the components, there is a “blind” region of about 20° between the emitter and detector, schematized as a pink-shaded area in Fig. S2b (Supporting Information), which is not accessible to measurements.

For each configuration, an unpatterned metallic reference is also characterized, in order to calculate the RCS ratio in Eq. (4).

A more detailed description of the experimental setups, as well as the data processing, is provided in the Supplementary Information. Here, we limit ourselves to highlight that the use of collimating planar lenses for both the emitter and receiver, and of a simple goniometric guiding trail for the receiver antenna allows to reach an unprecedented angular resolution (1°) for both the impinging and scattered signals, by comparison with previous schemes based mostly on off-axis parabolic mirrors^9–12,15^, where the precise determination of the incident and detection angles is more complex.

### Data availability

The datasets generated during and/or analyzed during the current study are available from the corresponding authors on reasonable request.

## Electronic supplementary material

Supplementary information
