# Supplementary material for: Suboptimal Coding Metasurfaces for Terahertz Diffuse Scattering
Source: Sci Rep. 2018 Aug 9;8:11908. doi: 10.1038/s41598-018-30375-z (PMC6085296; doi:10.1038/s41598-018-30375-z)
Supplement: Supplementary file 1 — Supplementary information [file 41598_2018_30375_MOESM1_ESM.pdf]

Supplementary Information for: Suboptimal Coding Metasurfaces for Terahertz Diffuse Scattering

Massimo Moccia [1,+], Can Koral [2,+], Gian Paolo Papari [2,3], Shuo Liu [4], Lei Zhang [4], Rui Yuan Wu [4], Giuseppe Castaldi [1], Tie Jun Cui [4,*], Vincenzo Galdi [1,*] , and Antonello Andreone [2,3,*]

[1] Fields & Waves Lab, Department of Engineering, University of Sannio, I-82100 Benevento, Italy
[2] Department of Physics, University of Naples “Federico II”, and CNR-SPIN, I-80125 Naples, Italy
[3] INFN Naples Unit, via Cinthia, I-80126, Naples, Italy
[4] State Key Laboratory of Millimeter Waves, Southeast University, Nanjing 210096, China

*Correspondence and requests for materials should be addressed to TJ.C. (tjcui@seu.edu.cn), V.G. (vgaldi@unisannio.it), or A.A. (andreone@unina.it)

[+]these authors contributed equally to this work

Details on Metasurface Design

Table S1 shows the coding sequences pertaining to the three designs, from which the 2-D binary codings are obtained via dyadic products. Figure S1 shows the resulting layouts. As mentioned in the main text, the P_5- and Q_5-type sequences consist of 32 bits, and the corresponding patterns (Figs. S1b and S1c, respectively) are *globally aperiodic*. Note that the first halves of these sequences coincide, whereas the second halves are flipped. Conversely, the P_2-type sequence is only four-element long, and the corresponding 2-D pattern is a periodic replication of this basic coding (see Fig. S1a) so as to obtain the same size as the other two.

Table S1. Coding sequences pertaining to the GRS P_2-, P_5- and Q_5-type designs.

Type	N	Coding sequence
P_2	4	1110
P_5	32	11101101111000101110110100011101
Q_5	32	11101101111000100001001011100010

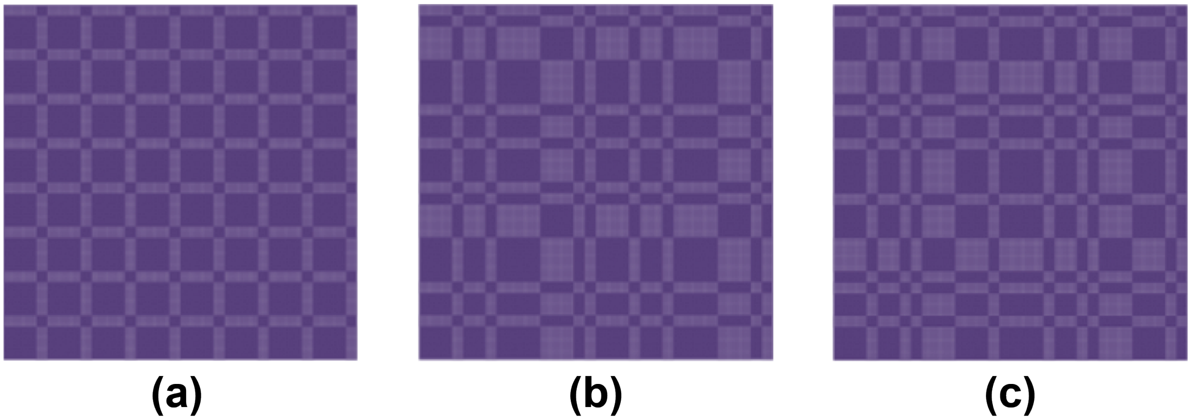

Figure S1. (**a**), (**b**), (**c**) 2-D patterns pertaining to the GRS P_2-, P_5- and Q_5-type designs, respectively. The codings are generated via dyadic products of the sequences in Table 1.

Details on Measurement Setup

Figure S2 illustrates the schematics of the two setups utilized for the experimental characterization of the metasurfaces (backscattering and angular-scan measurements).

A rendering of the customized THz time-domain spectrometer (TERA 15, MENLO Systems GmbH, Germany) is shown in Fig. S3a. The system is driven by a femtosecond fiber laser (operating at 1560 nm) with a fiber-coupled output with optical power < 100 mW, pulse length < 90 fs, and bandwidth ~3.5 THz. The setup employs fiber-coupled photoconductive antenna (PCA) modules (TERA 15 TX/RX FC) both for signal emission and detection. Best values for the antenna performance are: spectral range > 4 THz, dynamic range > 75 dB, and frequency resolution < 1.2 GHz.

Plano-convex, aspherical, polymethylpentene (TPX) lenses are used to collimate the broadband THz radiation onto both the metasurface and the unpatterned gold plated substrate used as a reference. The beam waist, measured via a standard edge technique, is found to be ~11 mm in diameter at 1 THz. A fiber-coupled opto-mechanical light path is utilized for the optical delay. For backscattering measurements (see the discussion below), a high transmittance single-pass silicon THz beam-splitter placed at 45° angle is utilized. The target holder and the beam-splitter are placed on kinematic mounts connected to a micro-scaled rotation stage to adjust the tilts. Accurate target positioning is maintained by a computer-controlled *x*-*y* stage. Since the spot size of the THz beam is slightly larger than the

metasurface dimensions (9.6×9.6 mm^2), a "wet masking" method is utilized to avoid any signal interference from the environment. Basically, the ends of the sample under test are covered with highly absorbing stripes (wet anti-dust cloths) in order to suppress any unwanted specular reflections from the surrounding unpatterned gold; these latter would be dominant at the specific incident angles (0, 10° or 20°) by comparison with the signal scattered by the metasurface.

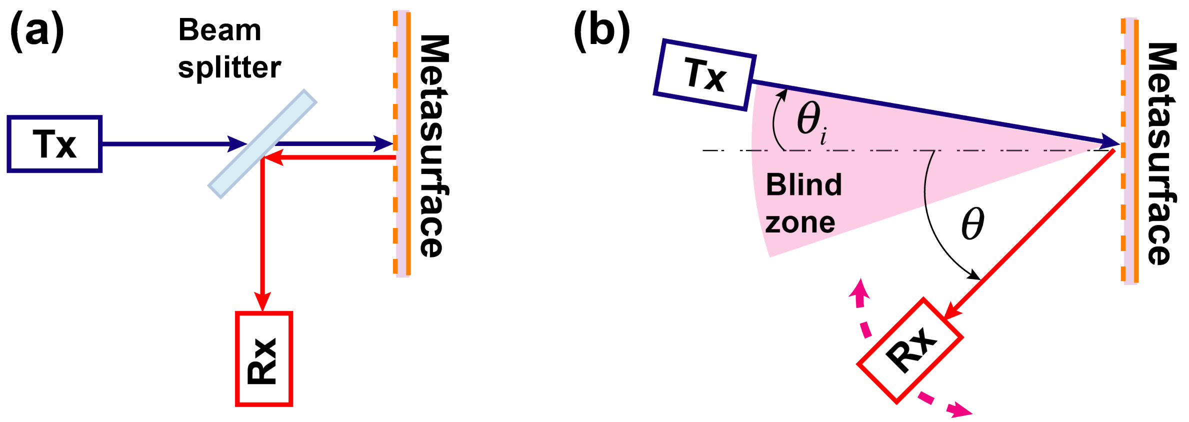

Figure S2. Schematics (not in scale) of the measurement setups. (**a**) Backscattering measurements. (**b**) Oblique-incidence and angular scan. The pink-shaded area represents an angular region that is not accessible by the receiver (blind zone).

As mentioned in the main text, two different setups are utilized. Figure S3b shows a rendering of the setup for backscattering measurements (also schematized in Fig. S2a). In this configuration, the THz signal emitted by the first PCA impinges directly onto the target at normal incidence after passing through the silicon beam-splitter, and the backscattered signal is collected along the orthogonal direction.

Figure S3c shows instead the rendering of the setup for angular measurements (also schematized in Fig. S2b). In this case, the THz beam impinges on the target with a fixed angle (0, 10°, 20°), without passing through the beam-splitter. The signal scattered by the metasurface is collected along a circular path, with angular step of 1°, by manually changing the position of the detector on a goniometer. Note that the physical dimensions of the optical components prevent the full overlap of the emitter and detector arms; this creates a "blind" region of about 20° between the two PCAs. Moreover, since the required manual scanning prevents the possibility to operate at low-pressure conditions, the measurements are affected by the water-vapor absorption peaks (as clearly observable in Figs. 3-5 and S4-S6).

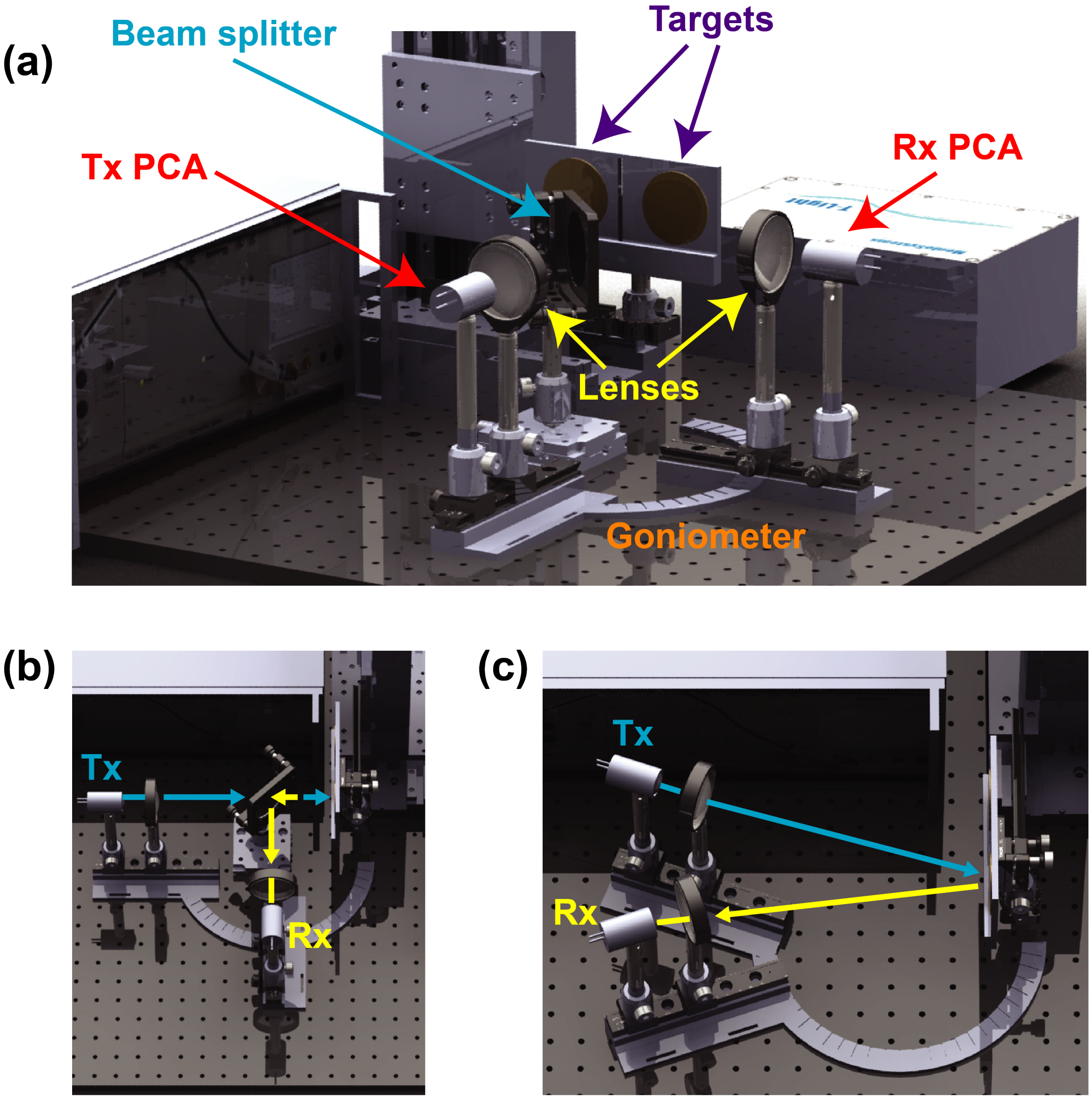

Figure S3. (**a**) Rendering of the customized THz time-domain spectrometer. (**b**), (**c**) Rendering of the backscattering and angular-scan setups, respectively (cf. the schematics in Fig. S2).

For both configurations, time-domain data are acquired with 100 ms integration time, and then converted to the frequency domain via fast Fourier transform (FFT) with ~4 GHz resolution. Data acquisition is realized by means of a lock-in amplifier coupled with in-house electronics and computer software. It is worth highlighting that, although the working bandwidth of the system is 0.2-3.5 THz, our observations are focused on the frequency region 0.8-1.6 THz where the response of the two basic unit-cells is close to the desired 180° out-of-phase condition (cf. Fig. 2a).

Results for GRS Q_5-type Design

Figures S4-S6 illustrate the results pertaining to the GRS Q_5-type design. Overall, the experimental results are comparable with those observed for the P_5-type design (cf. Figs. 3b, 5, 6c, 6d). It is worth stressing that the theoretical predictions for the Q_5-type design are rather extreme, as the specular reflection should be exactly zero at those frequencies where the reflection coefficients of the two elements are out of phase. As it can be observed from Fig. 2a, this condition is numerically met at the design frequency of 1 THz and also at a higher frequency around 1.3 THz. Accordingly, the numerically predicted RCS ratio attains two nulls at those frequencies (see Fig. S4), and exhibits generally smaller values than the P_5-type design (cf. Fig. 3b) over most of the observed frequency region. On the other hand, the measured backscattering is not very different from the P_5-type case, with a visible dip at the design frequency. The discrepancies with the numerical simulations at higher frequencies can be attributed to the various approximations in the model, as well as fabrication tolerances and uncertainty in the constitutive parameters of the dielectric layer. Moreover, we estimate that reductions in the RCS ratio >40 dB are likely beyond the experimental sensitivity of our current measurement setup. Nevertheless, we remark that RCS reductions ~20 dB are maintained over a sizable bandwidth.

From Fig. S5, we observe a rather flat angular response, uniformly across the observed frequency region, once again devoid of Bragg-type peaks. In fact, unlike the P_5-type case (cf. Fig. 5b), the measured angle/frequency map in Fig. S5b does not show any visible residual of trivial Bragg modes. Also remarkable is the good agreement between simulations and measurements in the angular response at the design frequency of 1 THz (Fig. S5d).

Finally, Fig. S6 shows the results for oblique incidence. Also in this case, the experimental responses are in line with those observed in the P_5-type case (cf. Figs. 6c and 6d), with the RCS generally exhibiting a reduction ≥ 20 dB. Once again, the perceived deterioration of the agreement with the simulations is attributable to the various approximations and inaccuracies in our modeling (e.g., unmodeled finite-size and coupling effects) which, as already mentioned in the main text, become more prominent at oblique incidence.

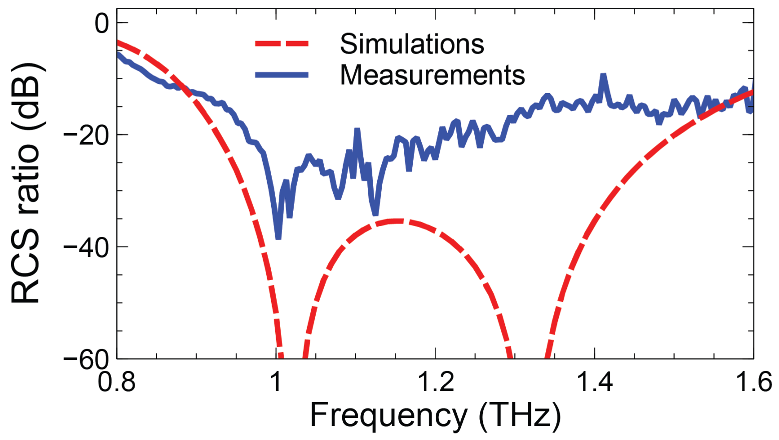

Figure S4. Measured (blue-solid) and simulated (red-dashed) RCS ratio [Eq. (4)] for normal-incidence and backscattering-direction ($\theta = \theta_i = 0$, obtained with the setup in Fig. S2a), as a function of frequency, for the GRS Q_5-type design. The coding sequence and corresponding layout are explicitly given in Table S1 and Fig. S1.

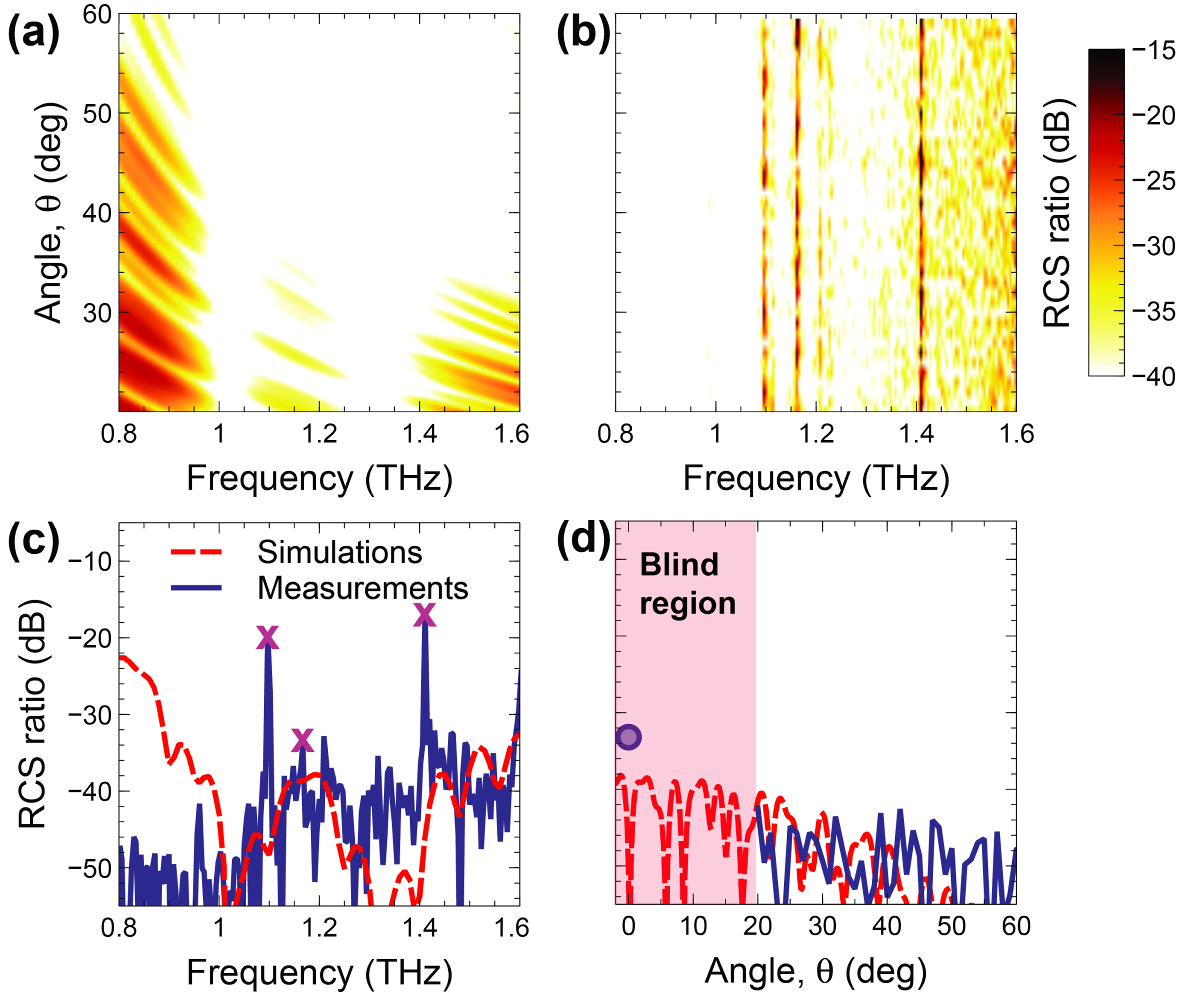

Figure S5. (**a**), (**b**) Simulated and measured, respectively, RCS ratio [Eq. (4)] in false-color scale, for normal-incidence, as a function of frequency and observation angle, for the GRS Q_5-type design. Measurements are obtained via the setup in Fig. S2a. (**c**), (**d**) Representative cuts at $\theta = 30°$ and $f = 1$ THz, respectively, comparing measurements (blue-solid) and simulations (red-dashed). The magenta-cross markers in panel (c) indicate the water-vapor absorption peaks. The pink-shaded area in panel (d) indicates a 20° angular region that is not accessible by the detector (blind region, cf. Fig. S2b). Also shown (blue-circle marker) is the backscattering measurement sample (extracted from Fig. S4).

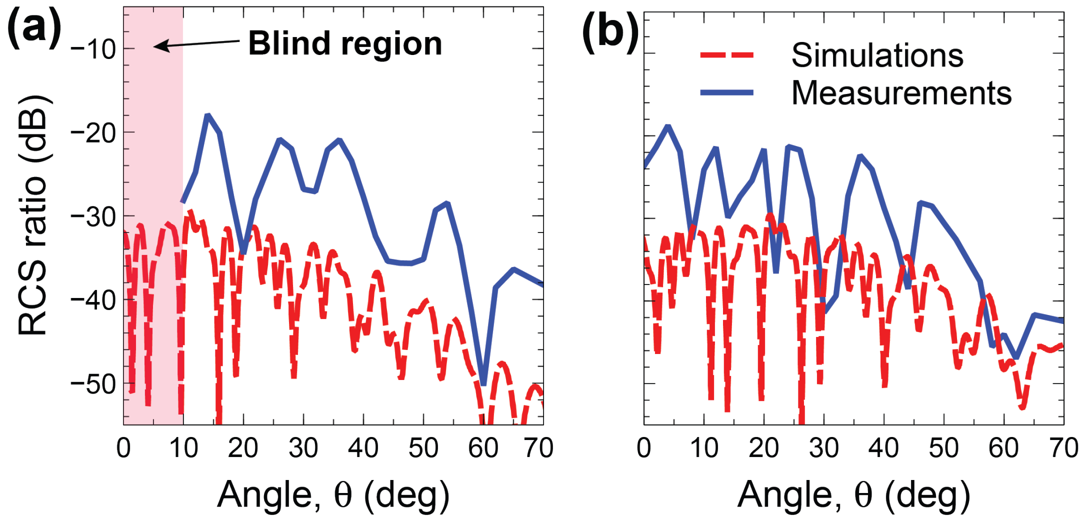

Figure S6. (**a**), (**b**) As in Fig. S5d, but for oblique incidence with $\theta_i = 10°$ and $20°$, respectively. Note that, for the chosen observation ranges, the blind region is 10° for $\theta_i = 10°$ and does not occur for $\theta_i = 20°$.
